# A Novel Method of Measuring Instantaneous Frequency of an Ultrafast Frequency Modulated Continuous-Wave Laser

**DOI:** 10.3390/s20143834

**Published:** 2020-07-09

**Authors:** Jiewei Yang, Tianxin Yang, Zhaoying Wang, Dongfang Jia, Chunfeng Ge

**Affiliations:** Key Laboratory of the Ministry of Education on Optoelectronic Information Technology, School of Precision Instrument and Optoelectronics Engineering, Tianjin University, Tianjin 300072, China; yang_jw@tju.edu.cn (J.Y.); wangzy@tju.edu.cn (Z.W.); jiadf@tju.edu.cn (D.J.); gechunfeng@tju.edu.cn (C.G.)

**Keywords:** frequency modulated continuous-wave (FMCW), light detection and ranging (LiDAR), instantaneous frequency, coherent optical spectrum analyzer (COSA), time-frequency curve

## Abstract

Ultrafast linear frequency modulated continuous-wave (FMCW) lasers are a special category of CW lasers. The linear FMCW laser is the light source for many sensing applications, especially for light detection and ranging (LiDAR). However, systems for the generation of high quality linear FMCW light are limited and diverse in terms of technical approaches and mechanisms. Due to a lack of characterization methods for linear FMCW lasers, it is difficult to compare and judge the generation systems in the same category. We propose a novel scheme for measuring the mapping relationship between instantaneous frequency and time of a FMCW laser based on a modified coherent optical spectrum analyzer (COSA) and digital signal processing (DSP) method. Our method has the potential to measure the instantaneous frequency of a FMCW laser at an unlimited sweep rate. In this paper, we demonstrate how to use this new method to precisely measure a FMCW laser at a large fast sweep rate of 5000 THz/s by both simulation and experiments. We find experimentally that the uncertainty of this method is less than 100 kHz and can be improved further if a frequency feedback servo system is introduced to stabilize the local CW laser.

## 1. Introduction

An ultrafast linear frequency modulated continuous-wave (FMCW) laser in which the frequency changes continuously with time in a periodic fashion while its intensity is kept constant is extensively required in various applications, such as measurements for absolute distance, speed, and vibration [1,2,3], light detection and ranging (LiDAR) [4,5], coherent optical spectrum analyzer (COSA) [6,7,8], optical coherence tomography (OCT) [9,10,11], micro-cavity dispersion measurement [12], satellite formation flying [13], precision manufacturing [14], and so on. Many applications ideally need a linear FMCW light source with broad frequency sweep excursion, narrow instantaneous linewidth, fast frequency sweep rate, and excellent frequency modulation linearity (without mode-hopping) [15,16,17], so that a FMCW LiDAR could detect remote targets at ultrafast scanning rates with ultrahigh range resolution compared to its counterpart, a time-of-flight (ToF) LiDAR.

Among these characteristics, the frequency sweep rate is a key parameter for the application of a real-time optical sensing system in which a FMCW laser is embedded. The responsivity of the whole sensing system is related to the frequency sweep rate of the FMCW laser, while other parameters, including the linewidth and sweep excursion, are also important. For example, in manufacturing applications the inspection update rate is often more important than accuracy or precision.

Although it is challenging to simultaneously realize submicrometer accuracy (and/or precision) and high update rates in a remote sensing system, as in a linear FMCW LiDAR, both are required in the application of tight-formation flying of satellites, for which the ranging accuracy is required to reach the nanometer level [13]. Based on the principle of linear FMCW LiDAR, the detection range of z is governed by Equation (1) [18].

(1) $$z=\left(\frac{c}{2\gamma }\right)\cdot \Delta f_{FMCW}$$

where c is the speed of light, γ is the frequency sweep rate, and $\Delta f_{FMCW}$ is the frequency difference between the returning light from the remote target and a local reference light which is just emitting out from the linear FMCW laser. From Equation (1), the uncertainty of the detection range δz obeys Equation (2).

(2) $$\delta z=\bar{z}\left(\frac{\delta \gamma }{\bar{\gamma }}+\frac{\delta \Delta f_{FMCW}}{{\bar{\Delta f}}_{FMCW}}\right)$$

In Equation (2), we can see that, for detecting a long distance ($\overset{¯}{z}$ is large) accurately, the two terms in the parenthesis should be as small as possible. So, it is obvious that a faster frequency sweep rate $\overset{¯}{\gamma }$ is preferred for the sake of higher range accuracy. Normally, the frequency sweep rate $\overset{¯}{\gamma }$ is above THz/s over a broad optical bandwidth of several THz so that no general optical spectrum analyzer or electronic spectrometer could be able to catch up with the sweep CW light. Neither instrument could obtain the instantaneous frequency over the broad sweep excursion of several THz. A dedicated method which was able to measure the instantaneous frequency of a linear FMCW laser over a sweep excursion of 5 THz was proposed and completed by a group at the National Institute of Standards and Technology (NIST) in the United States [19]. They successfully retrieved the phase curve versus time from their experimental data when an under-test linear FMCW laser was swept against a one femtosecond mode-locked frequency comb in the well-known NIST dual-comb system [20]. Then, the time-frequency curve of the FMCW laser, i.e., the instantaneous frequencies versus time during the whole sweep period, was obtained by simply calculating a numerical first-order derivative with time of the phase curve they just retrieved. However, they encountered a technical problem in which the phase was ambiguous at the half way point between two adjacent comb teeth, because the in-phase/quadrature (IQ) demodulator that they used in their measurement system could not identify two different situations at the middle point of two comb teeth, i.e., the IQ detectors cannot judge whether the sweep is approaching to an individual tooth or moving away from the tooth. Therefore, the time-frequency curve became ‘corrupted’ at the middle of two adjacent comb teeth. That meant that the phase at that moment could not be retrieved from the data which were collected by the IQ demodulator. One year later, they overcame the problem technically by removing the ‘corrupted’ parts in two measurements with frequency-shifted combs and stitching the rest of the ‘good parts’ together to obtain a smooth phase curve versus time. So, they claimed that the maximum measurable frequency sweep rate was up to 1500 THz/s using the comb system for which the frequency spacing was about 100 MHz [21]. Very soon after this, the record was updated to 3400 THz/s by doubling the comb spacing to 200 MHz [2].

## 2. Principles for Measuring Instantaneous Frequency of an Under-Test FMCW Laser at Fast Sweep Rate

NIST’s dual-comb system might be the most elaborate system for optical frequency measurement in the world [2,20,21,22,23,24], however in principle, it might not be the best choice for directly measuring the instantaneous frequency of a FMCW light, especially when the frequency sweeps at a fast rate. Because, according to the definition of frequency, a measuring time window has to be specified before the frequency measurement is initiated, then how many periods in this measuring time window is counted by the fact that, in one period, the optical phase changes by 2π. For a CW laser with a constant frequency or wavelength, there is a rule that the longer the time window is, the smaller the frequency uncertainty that can be observed. However, for a FMCW laser with a high sweep rate, the rule does not hold anymore. Because the frequency of the FMCW laser changes dramatically during a long-time window, the instantaneous frequency cannot be defined well. Theoretically, according to the exact definition of instantaneous frequency for a frequency sweep laser, the measuring time window should be zero. Obviously, this is impossible to realize in NIST’s system. So, the instantaneous frequency was measured in a very short time window with a tolerance uncertainty. It was found experimentally in NIST’s paper that the accuracy was improved only by one order of magnitude while the length of the measuring time window had to be increased by two orders of magnitude. So, they claimed that their system was suitable for the FMCW laser where the sweep rate was less than 3400 THz/s [2].

In this paper, we avoid the issue of the measuring time window and propose a new scheme for measuring the instantaneous frequency of a FMCW laser at a fast sweep rate against a CW optical frequency comb rather than a traditional mode-locked femtosecond pulsed optical frequency comb. The CW optical frequency comb here actually is a single-longitudinal-mode multi-wavelength laser. It could be an electro-optic optical frequency comb generated by a recirculating frequency-shifted optical fiber loop based on a technique that uses an external modulation of a seed CW laser [17,25], or it could be generated by an optical fiber CW laser that has a Fabry–Perot comb filter inserted in the ring cavity [26]. In short, there is no fixed phase relationship among these individual comb teeth so that each comb tooth can be seen as a CW light wave with a single frequency component. Therefore, before the CW comb is used to measure the instantaneous frequency of a FMCW laser, the frequency or wavelength of each comb tooth can be known by measuring them precisely in a long time window using a general advanced optical spectrum analyzer which is available in the marketplace.

Assuming that the known absolute optical frequencies of the CW comb are labeled as a set of numbers in Hertz, noted as $\left\{\nu_{1},\nu_{2},\nu_{3},\ldots ,\nu_{n}\right\}$, where $\nu_{n}$ is the known frequency of the *n*th tooth and $\nu_{n}=\nu_{1}+\left(n-1\right)\cdot \Delta \nu$, where Δν is the frequency spacing of the CW comb, and the span of the CW comb $\left(\nu_{n}-\nu_{1}\right)$ should cover the full sweep excursion of the under-test FMCW laser. Now, let another set $\left\{t_{1},t_{2},t_{3},\ldots ,t_{n}\right\}$ represent the times of the moments when the under-test FMCW laser sweeps across each CW comb tooth which frequencies are known as $\left\{\nu_{1},\nu_{2},\nu_{3},\ldots ,\nu_{n}\right\}$ in the frequency domain. It is obvious that there is a one-to-one map between the two sets. Each element in the time set $\left\{t_{1},t_{2},t_{3},\ldots ,t_{n}\right\}$ can be determined by the method we present in this paper. By our method, *n* number of instantaneous frequencies of the under-test FMCW laser can be obtained in the form of $\left\{\nu_{1}\left(t_{1}\right),\nu_{2}\left(t_{2}\right),\nu_{3}\left(t_{3}\right),\ldots ,\nu_{n}\left(t_{n}\right)\right\}$, then the time-frequency curve of the FMCW light over the full sweep excursion can be depicted. This is the principle we propose for measuring the instantaneous frequency of a FMCW light. Based on this principle, we propose a new scheme for measuring the instantaneous frequency of a FMCW light wave using a CW frequency comb by building a one-to-one mapping between the known frequencies of the CW comb tooth and points in time of a FMCW light wave sweeping across these CW comb tooth.

In this paper, a basic technique for determining the point in time of a FMCW light wave sweeping across one tooth of the CW comb is presented by both simulation and experiments. The heterodyne signal between the under-test FMCW light source and a local stable CW laser with an accurately known frequency or wavelength is recorded by a pair of balanced photodetectors connected to an advanced broadband real-time oscilloscope (OSC). In order to precisely identify the time of the moment when the under-test FMCW light just swept across the local stable CW laser in the frequency domain, the power of the local stable CW laser is amplified so that the central part of heterodyne signal can be chopped off slightly due to the saturation effect of the balanced photodetectors. However, it would not reduce the accuracy for us to determine the point in time because we developed a new technique to deal with the problem. By taking a digital signal processing (DSP) of a numerical low-pass filtering of the recorded heterodyne signal, the well-defined profile of the interference pattern can be retrieved clearly. This profile should be centered at the time of the moment when the instantaneous frequency of the FMCW laser is equal to the frequency of the local CW laser which is already accurately known. The time at the center of the interference pattern can be determined precisely by fitting the profile numerically. Therefore, based on the new technique, the one-to-one mapping between the time and the instantaneous frequency of the FMCW laser could be achieved in this way if the CW laser was replaced by the CW frequency comb that we just described in former paragraph.

In this paper, a linear FMCW light is generated with a sweep excursion of 20 GHz in a period of 4 μs, so the sweep rate can reach to 5000 THz/s. Using the new technique, we developed, one instantaneous frequency of the fast sweep FMCW light can be accurately measured at one instant of time. It is shown by our experiment that the uncertainty of the measured instantaneous frequency is less than 100 kHz.

## 3. Generation of the Under-Test FMCW Light and Its Time-Frequency Curve Measured by an OSC

**Figure 1:**
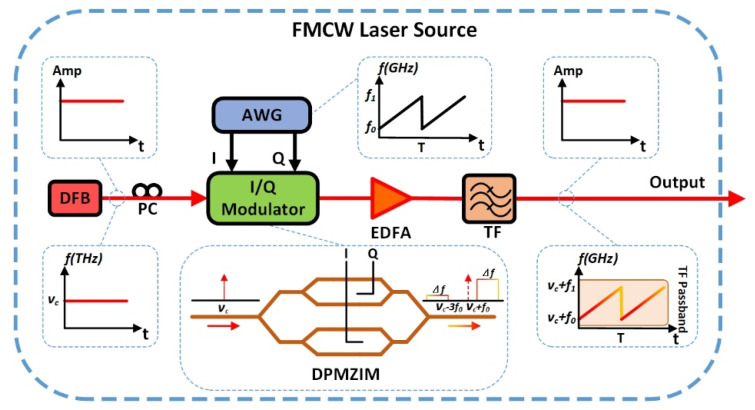
The generation system of the under-test FMCW light wave.

The under-test FMCW light wave is generated by an ultra-fast swept laser system which is shown in Figure 1. The seed laser is a distributed feedback (DFB) semiconductor laser emitting out a CW light with narrow linewidth. The CW light is modulated by an in-phase/quadrature (I/Q) modulator which is driven by an arbitrary waveform generator (AWG). The I/Q modulator is a double parallel Mach–Zehnder interference modulator (DPMZIM) operating in single-sideband (SSB) mode. The AWG generates a linearly frequency swept signal with a period of *T* and it is divided into two branches, named as in-phase (I) branch and quadrature (Q) branch, feeding into the I input and Q input of the optical I/Q modulator, respectively. Based on the principle described in our previous paper [17,27], the linearly frequency swept CW light, which is actually the component of the +1 order side band, is generated from the I/Q modulator. The power of the linear FMCW light is then amplified by an erbium-doped fiber amplifier (EDFA), followed by a tunable optical filter to suppress the unnecessary residual high order sidebands such as the minus 3rd order side band. Finally, the linear FMCW light is generated and tested later in order to show how our measurement system works.

**Figure 2:**
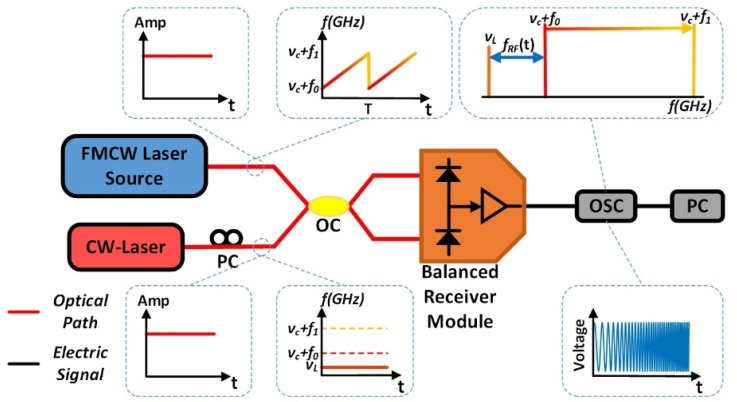
The heterodyne detection system of the under-test FMCW light wave.

The under-test FMCW light could be generated from the system shown in Figure 1. The frequency of the FMCW light linearly sweeps over an excursion of $\left(f_{1}-f_{0}\right)$ during a period of *T* from $\left(\nu_{c}+f_{0}\right)$ to $\left(\nu_{c}+f_{1}\right)$, where $\nu_{c}$ is the optical frequency of the seed DFB laser. To check the frequency sweep linearity of the FMCW light, we use a heterodyne detection system shown in Figure 2 to record a heterodyne signal that generates at an optical coupler (OC) by photonic mixing the linear FMCW light with a stable CW laser which is a tunable external cavity laser diode (TECLD). The optical frequency $\nu_{L}$ of the TECLD is tuned to be close to the sweep excursion of the FMCW light in the frequency domain, but fixed at a point on the left side of the sweep excursion in the frequency domain, i.e., $\nu_{L}<\left(\nu_{c}+f_{0}\right)$ (see the spectrum in the inset of Figure 2). Then, the time-frequency curve of the FMCW light generated in the system shown in Figure 1 can be obtained by an algorithm of short time Fourier transform (STFT) for example, using a personal computer (PC), based on the data collected by a pair of balanced photodetectors and a real-time OSC in the system shown in Figure 2.

The devices and equipment we used in our generation system (see Figure 1) and detection system (see Figure 2) are listed as follows: In Figure 1, the seed laser of the under-test FMCW light source is a DFB laser with a linewidth of about 20 MHz at the wavelength of 1550 nm. The electronic linear FMCW signal is generated from the AWG (Keysight, M8195A, sampling rate of 65 GSa/s) with peak-to-peak amplitude $V_{PP}$ = 1 V, a sweep excursion from $f_{0}$ = 5 GHz to $f_{1}$ = 25 GHz in a period *T* = 4 μs. So, the sweep excursion Δf is equal to 20 GHz and the sweep rate γ is equal to 5000 THz/s. In Figure 2 the CW laser (TECLD, Keysight, 81606A) with a linewidth of less than 100 Hz, output power of 12 dBm, is tuned carefully to let $\left(\nu_{c}-\nu_{L}\right)$ be equal to 5.0 GHz. The tunable optical filter (Santec-OTF-350, minimum bandpass of 0.1 nm, 12.5 GHz @1550 nm) is set to be centered at 1550 nm with an appropriate bandwidth. The pair of balanced photodetectors is centered at 1550 nm with a 3dB bandwidth of 40 GHz. A real-time OSC (Keysight, MSOV334A, bandwidth of 33 GHz) with a sampling rate of 80 GSa/s records and stores the data of the heterodyne signal. The STFT is applied to those data with a frequency uncertainty of about 50 MHz due to the limited size of the time window we selected in STFT calculation. Large frequency uncertainty is the weakness of the traditional method based on STFT calculations. We will compare it with our new method presented in this paper.

**Figure 3:**
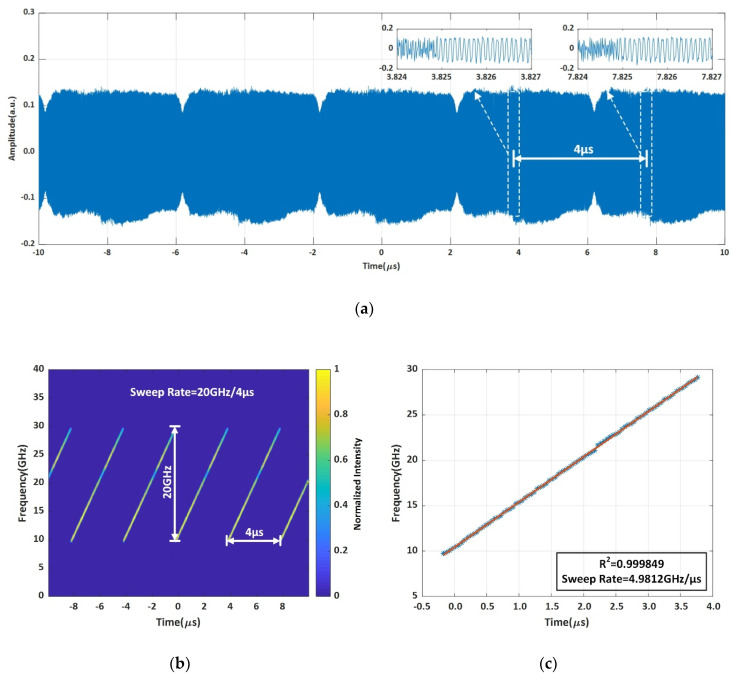
The under-test FMCW light wave. (**a**) The heterodyne signal, (**b**) multi-period time-frequency curve, (**c**) zoomed-in time-frequency curve in one period.

The measurement results are shown in Figure 3. The heterodyne signal between the FMCW light generated by the system shown in Figure 1 and a CW laser (TECLD) is recorded by the OSC (see Figure 2), and then plotted in Figure 3a. There are some intensity ripples over the 5 periods. These ripples may be caused by the imperfect responsivity over the broadband of 20 GHz of the balanced photodetectors. However, what we are concerned with is the frequency characteristics, such as the sweep linearity $R^{2}$ and sweep rate γ. The STFT curve of the heterodyne signal is shown as a time-frequency curve of the FMCW light in Figure 3b. By zooming-in the time-frequency curve in one period of 4 μs, it can be seen in Figure 3c that the sweep linearity $R^{2}$ = 0.999849 and sweep rate γ = 4.9812 GHz/μs = 4981.2 THz/s ≈ 5000 THz/s. It can be seen from the curve that the uncertainty of the instantaneous frequency of the FMCW light is about 50 MHz. This is because of the limited sampling rate of the OSC (although it is 80 GSa/s) and the limited time window we used in performing the STFT, which is mainly limited by time consuming computer calculations. However, the time window for performing the STFT cannot be significantly increased to reduce the uncertainty because we are dealing with a fast swept FMCW light. The longer the time window is, the worse the accuracy would be. We have to do a tradeoff between the sampling rate of the OSC and the window size of the time consuming STFT. So, an uncertainty of 50 MHz is the best result we could have at present for an ultra-fast FMCW light of 5000 THz/s by using the advanced real-time OSC with an ultrahigh sampling rate of 80 GSa/s.

## 4. Simulations of New Technique for Measuring the Instantaneous Frequency of Linear FMCW Light

**Figure 4:**
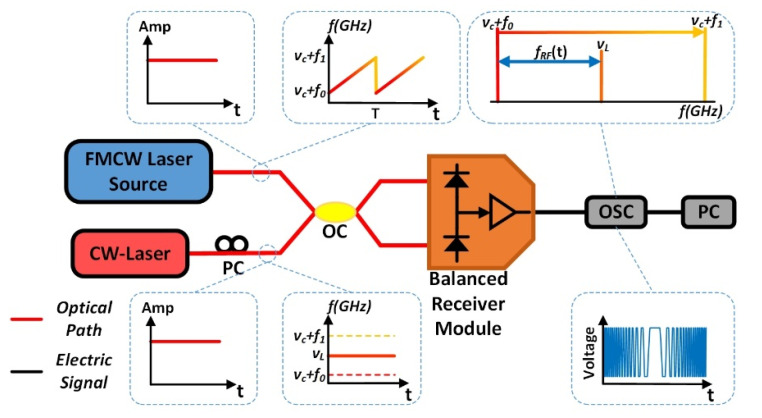
The measurement system of the under-test FMCW light wave.

The system for measuring instantaneous frequency of a FMCW light is shown in Figure 4. It may look like and resemble the configuration of a coherent optical spectrum analyzer (COSA) [6], however it is not. There are three differences from a COSA. First, the local laser here is a single frequency CW laser rather than a linear FMCW laser at a very slow sweep rate of 92 GHz/s in [6]. Second, the signal light to be tested here is the linear FMCW light at sweep rate of 5000 THz/s that can be generated by our system, as shown in Figure 1, and the parameter to be tested here is the instantaneous frequency of the fast sweep light, rather than a linewidth of a typical CW laser diode in Ref. [6]. Third, there is no electronic low-pass filter in front of the OSC in our system (see Figure 4), while in COSA there is one. It is necessary to have an electronic low-pass filter between the balanced photodetectors and the OSC to improve the frequency resolution of COSA. The smaller the bandwidth of the filter, the higher the resolution is. However, a very narrow bandwidth low-pass filter cannot work well here in our detection system. The reason that it would not work well is because our sweep rate of 5000 THz/s is too fast compared to 92 GHz/s in the original COSA [6]. Therefore, we need to perform numerical filtering to retrieve the interference pattern in a small band centered at the optical carrier frequency $\nu_{L}$ of our local CW laser.

**Figure 5:**
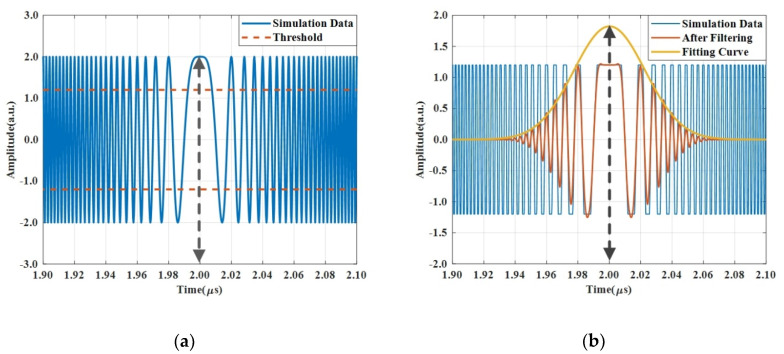
Simulation of the measurement method for determining the moment of time when the under-test FMCW light crosses the local CW laser in frequency domain. (**a**) The heterodyne signal collected by the OSC, (**b**) performing a numerical filtering and chopping off the top and bottom parts in the amplitude to eliminate ripple effects in amplitude.

The simulation results are shown in Figure 5. The interference pattern which is generated when the under-test FMCW light is swept over a non-filtered frequency band centered at the local CW laser in the frequency domain is recorded in real-time by an OSC and is presented in Figure 5a. Because both the FMCW light and the local single frequency laser are continuous waves with constant amplitude in an ideal simulation model, the interference signal should have a constant amplitude if there is no electronic low-pass filter in front of the OSC. However, because the FMCW light and the local CW laser are independent of each other in the time domain, the central part of the interference signal could present different shapes due to the various phase differences when two lights mix in the OC shown in Figure 4. Therefore, the data in the central part of the interference pattern is not reliable for retrieving the point in time when the FMCW sweeps across the point at the frequency of the local CW laser. Only the bilateral parts of the interference pattern which are formed when the FMCW is approaching to and leaving the local CW laser have consistency and are reliable. By performing a narrow-band numerical filtering operation, a profile of interference fringes can be clearly seen and are shown in yellow color in Figure 5b. The peak of the profile should be the point in time when the FMCW sweeps across the point at the frequency of the local CW laser. Thereby, it is easy to obtain the point in time precisely.

In reality, the interference signal which is shown in Figure 5a may be deformed in amplitude due to imperfect saturation effects of the balanced photodetectors. We can show that the imperfect effect does not interfere in recovering the profile of the interference pattern around the local CW laser. We deliberately performed an operation of chopping the top ‘head’ and bottom ‘foot’ parts of the interference signal along the dash lines shown in Figure 5a before the numerical filtering is taken. It can be seen in Figure 5b that the process of chopping off the ‘head’ and ‘foot’ just deforms parts of interference fringes. It does not change the shape and position of the interference profile in the time domain. In addition, this technique of chopping off the ‘head’ and ‘foot’ can increase the accuracy for determining the moment of time when the under-test FMCW sweep crosses the local CW laser in the frequency domain because the chopped interference signal becomes perfectly flat in amplitude so the symmetry of the profile can be improved. Furthermore, removing the ‘head’ and ‘foot’ parts can effectively reinforce the signals on left side and right side of the interference fringes which are shown in the orange color line in Figure 5b.

## 5. Experiments for Measuring the Instantaneous Frequency of a FMCW Light Source and Results Analysis

**Figure 6:**
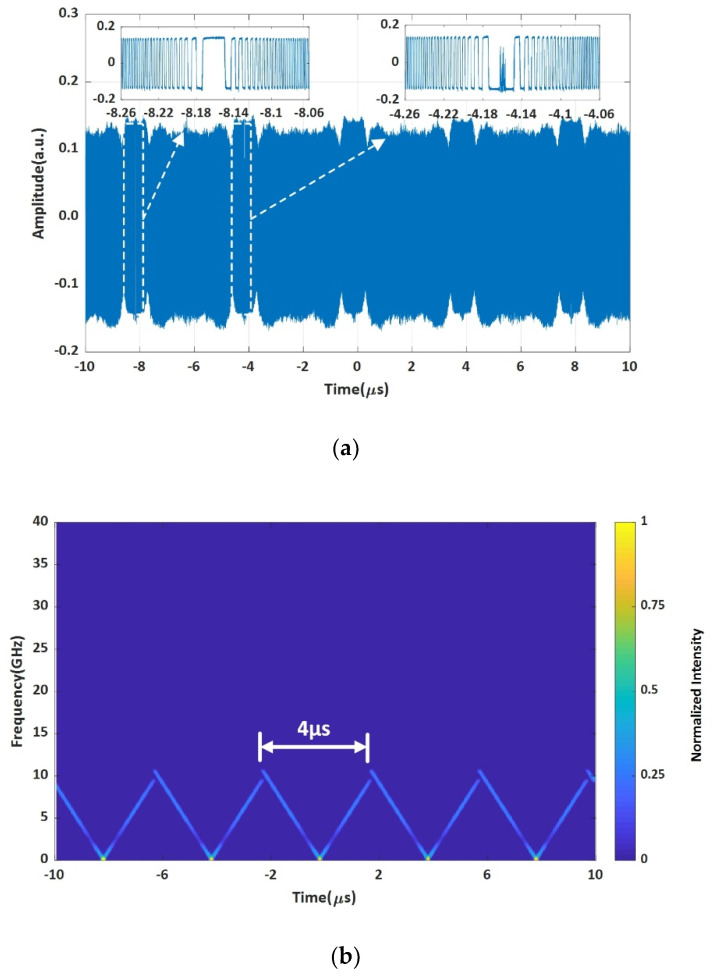
The experimental results for measuring the instantaneous frequency of a FMCW light source. (**a**) The heterodyne signal of the under-test FMCW light and a local CW laser. (**b**) The time-frequency curve of the heterodyne signal in (**a**) by the STFT algorithm.

The experimental setup is shown in Figure 4. The wavelength $\lambda_{L}$ (or frequency $\nu_{L}$) of the local CW laser (TECLD) is tuned carefully to be $\lambda_{L}$ = 1546.5033 nm to make sure that the local frequency $\nu_{L}$ is in the middle of the sweep excursion of the under-test FMCW light which has been generated in the system shown in Figure 1. The devices and equipment are the same as what have been described in previous sections. The heterodyne signal in 5 successive periods is recorded by the same real-time OSC for which the sampling rate is 80 GSa/s and is shown in Figure 6a. It can be seen that the heterodyne signals in amplitudes are not constant. However, the insets of the heterodyne signals are flatter. It can be seen in the two insets of Figure 6a that the central parts of heterodyne signals have different shapes. This is because there is no fixed phase relationship between the FMCW light and the local CW Laser (TECLD). Finally, it also can be seen clearly in the two insets that the saturation effects of the balanced photodetectors cut off the top and bottom parts of the heterodyne signals. We have discussed in previous sections that the saturation effect does not bring negative effects for precisely determining the point in time at the peak of an interference fringe profile. On the contrary, we found that chopping off the top and bottom parts could remove the ‘ripples’ in amplitude of the heterodyne signal making the profile fit better with the filtered interference fringes, and it does not shift the position in time of the profile.

Figure 6b shows the time-frequency curve based on the data in Figure 6a by using the algorithm of STFT. The turning point can be clearly seen in the middle of each period of 4 μs. Actually, the time of the turning point is the time coordinate we want to measure, however the accuracy for determining the turning point in time is not satisfied because the uncertainty of the STFT is about 50 MHz in Figure 6b. We have explained this issue in the previous sections for the traditional STFT method. We will show that our new method can reduce the uncertainty to less than 100 kHz in following paragraph.

**Figure 7:**
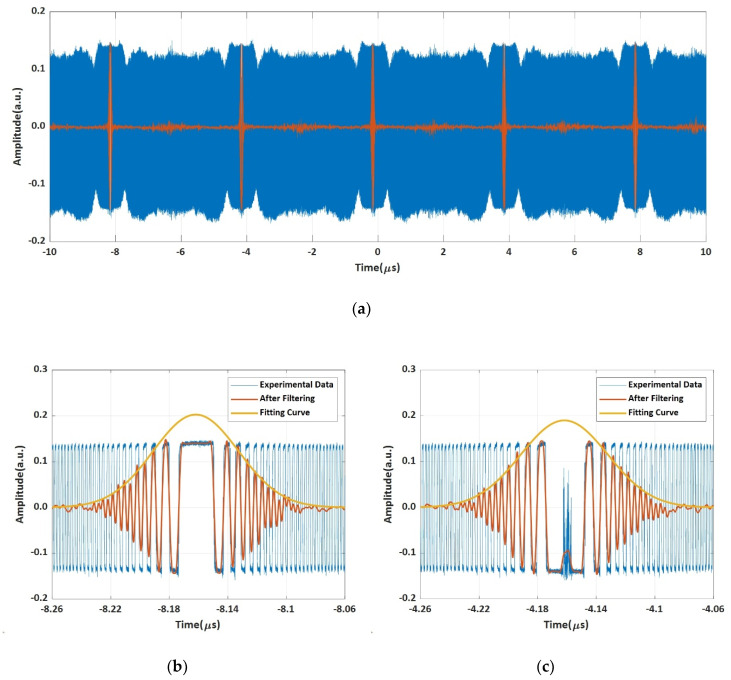
The multi-period heterodyne signal of the FMCW light and the local CW laser, (**a**) collected by the real-time OSA, (**b**) the 1st period, (**c**) the 2nd period.

We now show how we precisely determine the point in time when the FMCW sweeps across the local CW laser. The five-period heterodyne signal formed by the FMCW light and the local CW laser is collected by the real-time OSC and is shown as the blue curve in Figure 7a. We perform a numerical filtering to the data of the blue curve. The numerical filter is a finite impulse response (FIR) low-pass filter with a bandwidth of 600 kHz Kaiser type window with a stopband attenuation of 60 dB and transition zone steepness factor of 0.9999. The filtered curves are shown in orange color in Figure 7a. The curves in the first period and the second period in Figure 7a are zoomed-in and shown in Figure 7b,c, respectively. The central parts of the two interference fringes are different due to the reasons we have explained in previous section, however the profiles look the same as we have used the technique of chopping off the head and foot before performing numerical filtering. Now, it is straightforward to get five central points in time of these five profiles which are listed in Table 1.

The intervals of the 2nd profile, 3rd profile, 4th profile, and 5th profile to the 1st profile are listed in the third column in Table 1. They should be 0μs, 4.00000000 μs, 8.00000000 μs, 12.00000000 μs, and 16.00000000 μs if the AWG could be trusted to output signals exactly. Now the errors in time domain are obtained by subtracting these measured intervals with their ideal intervals, respectively. Then the equivalent instantaneous frequency errors (shown in the last column in Table 1) are obtained by multiplying the errors in time with the sweep rate of 5000 THz/s, respectively. It can be seen that the errors are increased by one order of magnitude from less than 100 kHz to around 1 MHz starting from the 4th profile. The reason could be the frequency drift of two lasers in our current system after 8 μs and the further drift after 12 μs. One is the local CW laser in the detection system shown in Figure 4, and the other is the seed DFB laser in the generation system of the linear FMCW light (see Figure 1). In our systems, we have not introduced optical frequency feedback servo systems to lock the local CW laser or the seed DFB laser to ultra-stable frequency reference sources so far [28,29,30]. If we had frequency feedback servo systems we believe that the uncertainty of this method for measuring the instantaneous frequency by one-to-one mapping time and frequency would be less than 100 kHz for which the accuracy can be increased by two orders of magnitude compared to 50 MHz obtained by other traditional methods.

## 6. Conclusions

In this paper, a new scheme is proposed for measuring the instantaneous frequency of a FMCW laser against a stable CW frequency comb over the full frequency sweep excursion of the FMCW laser. It is different from traditional methods based on femtosecond mode-locked pulsed frequency combs which measure the phase increment or phase decrement versus time in a short detection time window. However, the shorter the window is, the larger is the uncertainty of the frequency measurement. In our scheme, the issue of detection time window is avoided because we do not need to retrieve the phase function with time of the under-test FMCW laser, we just need to build a one-to-one mapping between the frequency of the each CW comb tooth and the point of time that the FMCW laser sweeps each comb tooth. Based on the one-to-one mapping, the time-frequency curve of the FMCW laser can be determined so that the instantaneous frequency of the FMCW laser over the full sweep excursion can be obtained precisely. We demonstrate how to determine the point in time that a FMCW laser sweeps one of the CW comb teeth by both simulation and experiments. It is shown by experiments that the uncertainty of measuring the instantaneous frequency of the FMCW at an ultrafast sweep rate of 5000 THz/s can reach less than 100 kHz, which is at least two orders of magnitude smaller in frequency compared to traditional methods. The method we developed in this paper has the potential to allow a precise characterization of an ultrafast sweep FMCW laser by combining a CW frequency comb with a large frequency spacing, such as 1 GHz, which is 10 times larger than traditional mode-locked pulsed frequency comb. So, the sweep rate of a FMCW laser could be potentially measured up to 10 times higher than 1500 THz/s while the uncertainty could be kept as low as 100 kHz, because measuring window issue in a traditional system can be avoided in our system in which instantaneous frequency is measured by a one-to-one mapping between the point in time of a FMCW laser and the known frequency of each CW comb tooth.

## Figures and Tables

**Table 1:** Central Points in Time of 5 Interference Profiles in Series.

Number of Profile	Center Point in Time of Profile	Interval to the Center of 1st Profile	Equivalent Frequency Error
1st	−8.16186146 μs	0 μs	0 kHz
2nd	−4.16184358 μs	4.00001788(0.00001788) μs	89.4 kHz
3rd	−0.16184241 μs	8.00001905(0.00001905) μs	95.25 kHz
4th	3.83792087 μs	11.99978233(−0.00021767) μs	−1.08835 MHz
5th	7.83911479 μs	16.00097625(0.00097625) μs	4.88125 MHz
